# Supplementary material for: Experimental visualization of water/ice phase distribution at cold start for practical-sized polymer electrolyte fuel cells
Source: Commun Eng. 2024 Feb 19;3:33. doi: 10.1038/s44172-024-00176-6 (PMC10955971; doi:10.1038/s44172-024-00176-6)
Supplement: Supplementary file 2 — Description of Additional Supplementary Files [file 44172_2024_176_MOESM2_ESM.pdf]

Description of Additional Supplementary Files

File name: Supplementary Movie 1

Description: Results of cold-start experiment.

File name: Supplementary Movie 2

Description: Results of forced-cooling experiment.
